# Combinatorial treatments of tamoxifen and SM6Met, an extract from *Cyclopia subternata* Vogel, are superior to either treatment alone in MCF-7 cells

**DOI:** 10.3389/fphar.2022.1017690

**Published:** 2022-09-22

**Authors:** Lorinda van Dyk, Nicolette J. D. Verhoog, Ann Louw

**Affiliations:** Department of Biochemistry, Stellenbosch University, Stellenbosch, South Africa

**Keywords:** *Cyclopia*, honeybush, tea extract, tamoxifen, combinatorial, synergism, breast cancer

## Abstract

Synergistic drug combinations are not only popular in antibiotic, anti-microbial, immune disease (i.e., AIDS) and viral infection studies, but has also gained traction in the field of cancer research as a multi-targeted approach. It has the potential to lower the doses needed of standard of care (SOC) therapeutic agents, whilst maintaining an effective therapeutic level. Lower dosages could ameliorate the fundamental problems such as drug resistance and metastasis associated with current SOC therapies. In the current study, we show that the combination of SM6Met with (2)-4-hydroxytamoxifen (4-OH-Tam, the active metabolite of tamoxifen) produces a strong synergistic effect in terms of inhibiting MCF7 ER-positive (ER^+^) breast cancer cell proliferation and that a 20 times lower dose of 4-OH-Tam in combination with SM6Met is required to produce the same inhibitory effect on cell proliferation as 4-OH-Tam on its own. Cell cycle analyses of the best combination ratios of SM6Met and 4-OH-Tam also suggests that the combination results in increased accumulation of cells in the S-phase and in the apoptotic phase. Moreover, the best combination ratio (20:1) of SM6Met with 4-OH-Tam displayed greater anti-metastatic potential in terms of inhibiting ER^+^ breast cancer cell migration, invasion, and colony formation than the SOC therapy alone, suggesting that SM6Met together with 4-OH-Tam could be a viable drug combination for not only delaying resistance and ameliorating the negative side-effects associated with current SOC therapies, like tamoxifen, but could also provide a novel, more affordable therapeutic alternative for treating or preventing ER^+^ breast cancer metastasis.

## 1 Introduction

Female breast cancer is the most frequently diagnosed cancer and the fifth leading cause of cancer death worldwide (Sung et al., 2021). However, death rates for female breast cancer are considerably higher in developing versus developed countries (Sankaranarayanan, 2011; Sung et al., 2021), which has been attributed to poverty and the high cost of cancer treatment. Moreover, severe side-effects and resistance to the current standard of care (SOC) hormone therapies have also proven to be significant issues in ER^+^ breast cancer treatment (Ziauddin et al., 2014; Costa et al., 2020). These factors have driven the investigation into combinatorial therapies (Almeida et al., 2020), many of which notably include the combination of SOC hormone therapies, such as tamoxifen, with natural products, such as tea leaf extracts (Chisholm et al., 2004; Yaacob et al., 2014; Ziauddin et al., 2014; Blasco-Benito et al., 2018; Khamis et al., 2018; Kim et al., 2020). Several of these studies have yielded promising synergistic anti-cancer effects (Samadi et al., 2014; Chisholm et al., 2004; Yaacob et al., 2014; Khamis et al., 2018; Kim et al., 2020), thereby providing scientific corroboration for a combinatorial treatment approach for breast cancer. Combinatorial treatments would be a more cost effective and safer alternative to SOC hormone therapies alone, as fewer side-effects are likely to occur, and the possibility of treatment resistance would be reduced.

Current SOC hormone therapies include selective estrogen receptor modulators (SERMs) and selective estrogen receptor down-regulators (SERDs). Both target the estrogen receptor (ER), which consists of an alpha (ERα) and beta (ERβ) subtype. ERα has been associated with sustained cell proliferation (and other cancer hallmarks) and ERβ has been linked to amelioration of ERα’s cancer promoting effects (Saji et al., 2000; Lazennec et al., 2001; Palmieri et al., 2002; Shaaban et al., 2003; Paruthiyil et al., 2004; Chang et al., 2006; Mfenyana et al., 2008; Visser et al., 2013; Zhou and Liu, 2020). SERMs act as either ER agonists or ER antagonists in a tissue selective manner. Tamoxifen, for instance, acts as an antagonist of ER in breast tissue, while acting as an ER agonist in the endometrium (Jordan, 2003; Vogel, 2018). The use of tamoxifen to prevent and treat ER^+^ breast cancer is due to its antagonistic effect in breast tissue (Dutertre and Smith, 2000; O’Regan and Jordan, 2002; Cuzick et al., 2013) and due to its effectiveness has been dubbed the gold standard of hormone therapy. However, tamoxifen is associated with adverse side-effects like hot flushes and blood clots (Jordan, 2004; Vogel, 2018) and some patients develop resistance to tamoxifen treatment (Chang, 2012; Osborne et al., 2013; Fan and Craig Jordan, 2014). The mechanism of resistance is still, however, not completely understood (Hayes and Lewis-Wambi, 2015; Ali et al., 2016; Yao et al., 2020), and SERDs, like fulvestrant, are used as a second line of treatment when tamoxifen resistance occurs (Chang, 2012). SERDs, stimulate proteasomal degradation of the ER upon binding to the ligand binding domain of the ER, thereby inhibiting ER signalling and estrogen binding (Dauvois et al., 1992, 1993). Fulvestrant is a pure anti-estrogen as it is an antagonist of both ER subtypes in all estrogen target tissues (ROBERTSON, 2002). Although fulvestrant, like tamoxifen, inhibits proliferation of ER^+^ breast cancer, it is associated with a wider range and increased severity of adverse side-effects such as hot flushes, muscle weakness, vasodilatation, asthenia, headache, back pain, nausea, vomiting, and diarrhoea (O’Regan and Jordan, 2002; Martínez Marín et al., 2009; Peng et al., 2009) limiting its use by patients who find the side-effects too severe to continue therapy.

Investigations into the mechanism of tamoxifen resistance has identified the ER as a valuable therapeutic target for overcoming tamoxifen resistance, suggesting that the addition of another ER-targeted anti-cancer agent in a combined therapy could prove effective in overcoming breast tumour resistance to tamoxifen (Riggins et al., 2007; Rondón-Lagos et al., 2016; Yao et al., 2020). SM6Met, a sequential methanol extract prepared from the indigenous fynbos plant, *Cyclopia subternata* Vogel (species of honeybush), was first identified as having an estrogenic potency comparable to many commercial phytoestrogenic nutraceuticals (Mfenyana et al., 2008), whereafter more recent studies showed its desirable ER-subtype selective activity by acting as an ERα antagonist and ERβ agonist (Louw et al., 2013; Visser et al., 2013), its inhibition of estradiol (E_2_)-induced ER^+^ breast cancer cell proliferation, its anti-inflammatory behaviour, and that it antagonizes E_2_-induced uterine growth (Visser 2013). Furthermore, *in vivo* studies have shown that SM6Met acts as chemopreventative agent against LA7-induced and *N*-Methyl-*N*-nitrosourea (MNU)-induced rat mammary gland carcinogenesis (Visser et al., 2016; Oyenihi et al., 2018) Therefore, SM6Met has the potential to modulate the ER via a complementary mechanism to that of the SOC hormone therapies, tamoxifen and fulvestrant, and thus in a combined therapy help delay resistance and ameliorate adverse effects (Tallarida et al., 1997; Louw, Ann; Joubert, Elizabeth; Visser, 2013).

The current study therefore evaluated the combinatorial potential of SM6Met to act synergistically with (2)-4-hydroxytamoxifen (4-OH-Tam), the active metabolite of tamoxifen, in preventing ER^+^ breast cancer proliferation. In addition, the effect of the best combination ratios of SM6Met and 4-OH-Tam on the redistribution of ER^+^ breast cancer cells within the phases of the cell cycle are investigated. Furthermore, the best combination ratios of SM6Met and 4-OH-Tam were assessed for their ability to inhibit ER^+^ breast cancer metastasis as evaluated through migration, invasion, and colony formation assays.

## 2 Materials and methods

### 2.1 Test panel

17β-Estradiol (E_2_) and (2)-4-hydroxytamoxifen (4-OH-Tam) were obtained from Sigma-Aldrich, South Africa. Dried plant material (leaves) of a *C. subternata* Vogel harvesting (M6; harvested on 30 March 2004 from a commercial plantation at Kanetberg farm near Barrydale, South Africa) was extracted according to a previously described procedure (Mortimer et al., 2015) and characterised (Supplementary Table S1) (Visser et al., 2013). 4-OH-Tam and E_2_ were prepared in absolute ethanol (EtOH), while SM6Met was prepared in dimethylsulfoxide (DMSO), which was diluted with EtOH to a final concentration of 25%. Test panel samples were further diluted in medium so that the final concentration of EtOH did not exceed 0.1% (v/v) and DMSO did not exceed 0.025% (v/v) when added to cells for treatment. E_2_ was used at 10^−11^M in all assays except when this concentration was below the sensitivity of the assay used, then 10^−9^M E_2_ was used.

### 2.2 Cell culture

The human MCF-7 BUS ER^+^ breast cancer cell line, received from Prof. Ana Soto (Tufts University, Boston), was maintained in culture medium comprised of Dulbecco’s Modified Eagle’s Medium (DMEM) containing 4.5 g/ml glucose (Sigma-Aldrich, South Africa) supplemented with 5% (v/v) heat-inactivated fetal calf serum (HI-FCS) (The Scientific Group, South Africa), 44 mM sodium-bicarbonate, 1 mM sodium-pyruvate and 100 IU/ml penicillin and 100 μg/ml streptomycin (1% penicillin-streptomycin (penstrep)) (Sigma-Aldrich, South Africa) as previously described (Szelei et al., 1997). All experiments were conducted within the first 35 passages since thawed from storage. Hoechst staining was routinely conducted to test for mycoplasma infection (Chen, 1977) and only mycoplasma-negative cells were used.

### 2.3 MTT cell proliferation assay

MTT (3-(4,5-dimethylthiazolyl-2)-2,5-diphenyltetrazolium bromide) cell viability assays were essentially conducted as described by Verhoog et al. (2007) with a few modifications. Briefly, the MCF-7BUS cells were withdrawn from steroids a week prior to plating by changing the culture medium to phenol red free DMEM supplemented with 5% fetal calf serum double stripped with dextran coated charcoal (DS-FCS), which was heat inactivated (DS-HI-FCS), and 1% penstrep, from here on referred to as treatment medium. Subsequently, on day one the MCF- 7BUS cells were seeded into 96-well tissue culture plates in treatment medium at a density of 3,000 cells/well and allowed to settle for 24 h.

#### 2.3.1 Combinatorial MTT assay

The cells were treated with 4-OH-Tam and SM6Met, 24 h after seeding, where increasing concentrations of SM6Met were combined with a constant concentration of 10^−9^M 4-OH-Tam (van Dyk, 2018) and increasing concentrations of 4-OH-Tam were combined with a constant concentration of 0.0098 μg/ml SM6Met in the presence of 10^−11^M E_2_ (See Supplementary Figure S1 for more detail). The cells were induced for a period of 7 days, wherein there were two retreatments on days three and six. On day eight, the cells were incubated with 1.25 mg/ml pre-warmed MTT solution for 4 h. The medium was removed and 200 μl DMSO was added to each well prior to an absorbance measurement at 550 nm on a BioTek^®^ PowerWave 340 spectrophotometer. Each assay included E_2_ as positive control and three negative solvent controls including 1) treatment medium, 2) 0.1% (v/v) EtOH in treatment medium and 3) 0.025% (v/v) DMSO in treatment medium. The results were expressed as fold proliferation relative to the positive control, E_2_, which was set at 1. See Supplementary Figure S2 for results from solvents and E_2._

#### 2.3.2 Fixed ratio isobologram analysis

The fixed isobologram method was performed as described by Tallarida *et al.* (Tallarida, 1992, Tallarida, 2002) to determine the interaction index (γ), which would indicate whether the combination of SM6Met with 4-OH-Tam is synergistic, additive, or antagonistic. In short, fixed ratio combination mixtures (of drug A (SM6Met) with drug B (4-OH-Tam) were prepared using the IC_50_ concentrations of each drug (3.128 × 10^–7^ μg/ml 4-OH-Tam and 8.841 × 10^–3^ μg/ml SM6Met). The chosen fixed ratios of SM6Met:4-OH-Tam included a 1:1, 1:5, 5:1, 1:10, 10:1, 1:20, 20:1, 1:50 and 50:1 ratio and the MCF- 7BUS cells were treated with a 2-fold dilution series of each ratio, all in the presence of 10^−11^M E_2_, creating dose response curves. The cells were induced for a period of 7 days, wherein there were two retreatments on days three and six. On day eight, the cells were incubated with 1.25 mg/ml pre-warmed MTT solution for 4 h. The medium was removed and 200 μl DMSO was added to each well prior to an absorbance measurement at 550 nm. Dose-response curves were obtained, and the concentration of each individual drug (i.e., SM6Met and 4-OH-Tam) was determined for each combination ratio at effect level 50%, 75% or 90% inhibition of ER^+^ breast cancer cell proliferation (shown in Table 1), which was used to determine the interaction index using the equation below:

$$\gamma =\frac{a}{A}+\frac{b}{B}$$

where γ is equal to the sum of the concentration of SM6Met [a] and 4-OH-Tam [b] at the IC_50_, IC_75_ or IC_90_ point of the selected fixed combination ratio, divided by the IC_50_, IC_75_ or IC_90_ concentration of (A) SM6Met alone (1:0) and (B) 4-OH-Tam alone (0:1). If the index is less than one (γ < 1) the combination is synergistic, if it is equal to one (γ = 1) the combination is additive and if it is greater than one (γ > 1) the combination is antagonistic. Each assay included E_2_ as positive control and three negative solvent controls including (1) treatment medium, (2) 0.1% (v/v) EtOH in treatment medium and (3) 0.025% (v/v) DMSO in treatment medium. The results were expressed as fold proliferation relative to the positive control, E_2_, which was set at 1. See Supplementary Figure S2 for results from solvents and E_2_.

**TABLE 1:** Summary of the concentrations (μg/ml) of SM6Met and 4-OH-Tam for each combination ratio, as determined by non-linear regression analysis, at effect levels of 50%, 75% and 90% in Figures 2A,B.

Ratio of SM6Met:4-OH-Tam	Concentration in μg/ml at the ED_50_ ± SD		Concentration in μg/ml at the ED_75_ ± SD		Concentration in μg/ml at the ED_90_ ± SD		Efficacy^c^
	SM6Met ( a ^a^)	4-OH-Tam ( b ^b^)	SM6Met ( a ^a^)	4-OH-Tam ( b ^b^)	SM6Met ( a ^a^)	4-OH-Tam ( b ^b^)	
1:0 (SM6Met alone)	1.66 × 10^–3^ ±0.23	-	9.95 × 10^–4^ ±0.61	-	4.97 × 10^–4^ ±0.94	-	16.4%
0:1 (4-OH-Tam alone)	-	5.32 × 10^–7^ ± 0.12	-	2.66 × 10^–7^ ± 0.34	-	1.25 × 10^–7^ ± 0.50	48.8%^**# # #**^ **^e^**
1:1	7.51 × 10^–3^ ± 0.12	2.66 × 10^–7^ ± 0.12	3.54 × 10^–3^ ± 0.33	1.25 × 10^–7^ ± 0.33	1.55 × 10^–3^ ± 0.50	5.47 × 10^–8^ ± 0.50	43.9%^**# # #**^
1:5	1.70 ± 0.14	6.01 × 10^–5^ ± 0.14	9.05 × 10^–1^ ± 0.28	3.20 × 10^–5^ ± 0.28	4.81 × 10^–1^ ± 0.44	1.70 × 10^–5^ ± 0.44	43.3%^**# # #**^
5:1	4.14 × 10^–4^ ± 0.11	1.47 × 10^–8^ ± 0.11	1.24 × 10^–4^ ± 0.21	4.40 × 10^–9^ ± 0.21	2.94 × 10^–5^ ± 0.35	1.04 × 10^–9^ ± 0.35	58%** **^d^** ^**# # #**^
1:10	9.62 × 10^–1^ ± 0.11	3.40 × 10^–5^ ± 0.11	4.53 × 10^–1^ ± 0.24	1.60 × 10^–5^ ± 0.24	1.98 × 10^–1^ ± 0.38	7.01 × 10^−6^ ± 0.38	48.5%^**# # #**^
10:1	2.21 × 10^–4^ ± 0.09	7.82 × 10^–9^ ± 0.09	7.60 × 10^–5^ ± 0.29	2.69 × 10^–9^ ± 0.29	2.59 × 10^–5^ ± 0.44	9.16 × 10^–10^ ± 0.44	54.6%^**# # #**^
1:20	2.55 × 10^–1^ ± 0.11	9.01 × 10^–6^ ± 0.11	8.49 × 10^–2^ ± 0.27	3.00 × 10^–6^ ± 0.27	3.01 × 10^–2^ ± 0.42	1.06 × 10^–6^ ± 0.42	57.9%^**# # #**^
20:1	1.31 × 10^–4^ ± 0.08	4.64 × 10^–9^ ± 0.08	4.83 × 10^–5^ ± 0.30	1.71 × 10^–9^ ± 0.30	1.55 × 10^–5^ ± 0.46	5.50 × 10^–10^ ± 0.46	54.9%* ^**# # #**^
1:50	1.20 × 10^–1^ ± 0.13	4.25 × 10^–6^ ± 0.13	6.01 × 10^–2^ ± 0.27	2.13 × 10^–6^ ± 0.27	3.01 × 10^–2^ ± 0.42	1.06 × 10^–6^ ± 0.42	62.1%^**# # #**^
50:1	2.76 × 10^–4^ ± 0.10	9.78 × 10^–9^ ± 0.10	9.67 × 10^–5^ ± 0.35	3.42 × 10^–9^ ± 0.35	2.24 × 10^−5^ ± 0.58	7.94 × 10^–10^ ± 0.58	58.4%** ^**# # #**^

a Variable representing the concentration of SM6Met used in the specified combination ratio that elicits the 50%, 75% or 90% inhibitory effect calculated from the dose response curve depicted in Figures 2A,B.

b Variable representing the concentration of 4-OH-Tam used in the specified combination ratio that elicits the half maximal 50%, 75% or 90% inhibitory effect calculated from the dose response curve depicted in Figures 2A,B.

c Efficacy shown as % inhibition of E_2_-induced proliferation.

d Statistically different from 4-OH-Tam alone—0:1 (* represents *p* < 0.05, ** represents *p* < 0.01 and *** represents *p* < 0.001).

e Statistically different from SM6Met alone i.e., 1:0 (^#^ represents *p* < 0.05, ^# #^ represents *p* < 0.01 and ^# # #^ represents *p* < 0.001).

### 2.4 Cell cycle analysis

MCF-7BUS ER^+^ breast cancer cells were plated into sterile 10 cm^2^ tissue culture dishes (Nest Biotechnology, China) at a density of 1 × 10^6^ cells/dish and allowed to settle for 24 h. After settling, the cells were serum starved for 4 hours by washing the cells once with 10 ml sterile, pre-warmed PBS per plate and replacing the medium with un-supplemented phenol red free DMEM. Thereafter, the medium was changed to the treatment medium (phenol red free DMEM supplemented with 5% DS-HI-FCS and 1% penstrep) and treated with the test panel (concentrations indicated in figure legend) for 48 h. After the treatment period the nuclei were isolated and stained using propidium iodide (PI) according to the instructions of the manufacturer of the CycleTEST^TM^ PLUS DNA reagent kit (Bectib Dickinson, South Africa). A 448 nm solid state sapphire laser was used to excite the PI-stained nuclei and emittance was measured in the PE Texas Red channel on a linear scale using a 610/20 bandpass filter. Histograms were generated of the fluorescent light emitted from the nuclei between 600–620 nm using the BD FACS Aria Cell sorter from Becton Dickinson (United States), and the FACS Diva 6.1.3. software. ModFit LT^TM^ 3.0 software (Verity Software House, United States) was used to analyse the fluorescence histograms to determine cell cycle phase distribution. Results were presented as bar graphs of the average percentage of cells in each cell cycle phase.

### 2.5 Scratch-wound healing assay (migration)

MCF-7BUS cells were seeded (1 × 10^6^ cells/well) into 12 well tissue culture plates and allowed to reach 100% confluency. After which the medium was changed to phenol red free DMEM supplemented with 5% DS-HI-FCS and 1% penstrep (treatment medium). Mytomycin C (5 μg/ml) was added to each well to inhibit cell proliferation and incubated for 2 hours. The mixture was then aspirated, and a “scratch” was introduced by scraping a vertical wound through the cell monolayer using a sterile 200 μl pipette tip, after which the cells were washed twice with 400 μl sterile, pre-warmed PBS. Treatment medium containing the test panel (concentrations indicated in figure legends) was carefully added to each well to avoid detachment of additional cells. The images representing time point zero (T_0_) were immediately taken using an Olympus IX81 widefield inverted microscope and thereafter, images were taken at intervals of 24 h. The images were analysed by measuring the distance between the edges of the wound using ImageJ software (Version 1.49). The distance migrated (moved) was calculated by taking the distance migrated at T_72_ and subtracting it from the distance of the initial wound (T_0_) and dividing the answer by the distance of the initial wound (T_0_). Results were presented as fold change relative to the average results of the three negative solvent controls including 1) treatment medium, 2) 0.1% (v/v) EtOH in treatment medium and 3) 0.025% (v/v) DMSO in treatment medium, which was set at 1.

### 2.6 Cell invasion assay

The MCF-7BUS cells were seeded (1 × 10^6^ cells/dish) into 10 cm tissue culture dishes. Twenty-four hours after seeding medium was changed to treatment medium and allowed 24 h to settle. The number of invasive cells were determined using the CytoSelect™ 96-well invasion assay kit (Cell Biolabs, Inc) as described by the manufacturer. In short, cell suspensions were prepared in un-supplemented phenol red free DMEM containing the test panel (concentrations indicated in figure legend) and seeded (5 × 10^5^ cells/chamber) into a rehydrated 96 well membrane chamber plate. This was then placed into the feeder tray containing the chemoattractant (DMEM supplemented with 10% DS-FCS) and incubated at 37°C for 24 h. The membrane chamber plate was then removed from the feeder tray and placed into the harvesting tray containing the cell detachment solution were the cells that invaded through the membrane were dislodged from the bottom of the membrane. The cells were then lysed and stained with 4x Lysis Buffer/CyQuant^®^ GR dye (Invitrogen) for 20 min at room temperature after which the invasive cells were quantified by measuring fluorescence at 480 nm/520 nm using the Thermo Scientific™ Varioskan plate reader. Results were presented as fold change relative to the average results of the three negative solvent controls including 1) treatment medium, 2) 0.1% (v/v) EtOH in treatment medium and 3) 0.025% (v/v) DMSO in treatment medium, which was set at 1.

### 2.7 Soft agar colony formation

Soft agar assays were conducted as previously described by Perkins et al. (2017). In short, MCF-7BUS cells were mixed with phenol red free DMEM supplemented with 5% DS-HI-FCS, 1% penstrep and 0,6% agarose (Sigma-Aldrich) and plated (1.5 × 10^4^ cells/well) on top of a solidified layer of phenol red free DMEM supplemented with 5% DS-HI-FCS, 1% penstrep and 1% agarose in a 24 well plate. The top cell containing layer was allowed an hour to set at room temperature after which 1 ml of treatment medium consisting of phenol red free DMEM supplemented with 5% DS-HI-FCS and 1% penstrep, containing the test panel (concentrations indicated in figure legend) was added to the wells. Cells were re-induced and fed weekly for 4 weeks by carefully removing and adding new treatment medium containing the test panel (concentrations indicated in figure legend), without disturbing the cell containing layer. On day 28, the cells were stained overnight with 0.005% crystal violet made up in 10% EtOH (diluted with distilled water). Plates were placed on an illuminated background and photographs were taken, which were analysed using ImageJ software (Version 1.49) to determine the number of colonies formed. Results were presented as fold change relative to the average results of the three negative solvent controls including 1) treatment medium, 2) 0.1% (v/v) EtOH in treatment medium and 3) 0.025% (v/v) DMSO in treatment medium, which was set at 1.

### 2.8 Statistical analysis of data

Graphical presentations and statistical analysis were performed using GraphPad Prism^®^ version 5 (GraphPad Software). One-way ANOVA analysis of variance with Tukey’s multiple comparisons test as post-test was used to determine statistical significance of results. Statistically significant differences (*p* < 0.05) are indicated with letters (“a”, “b”, “c”, etc.). Figures are representative of at least two independent biological experiments and error bars represent the standard deviation (SD).

## 3 Results

### 3.1 SM6Met in combination with 4-OH-Tam is more effective at inhibiting E_2_-induced ER^+^ breast cancer cell proliferation than each on their own

**FIGURE 1:**
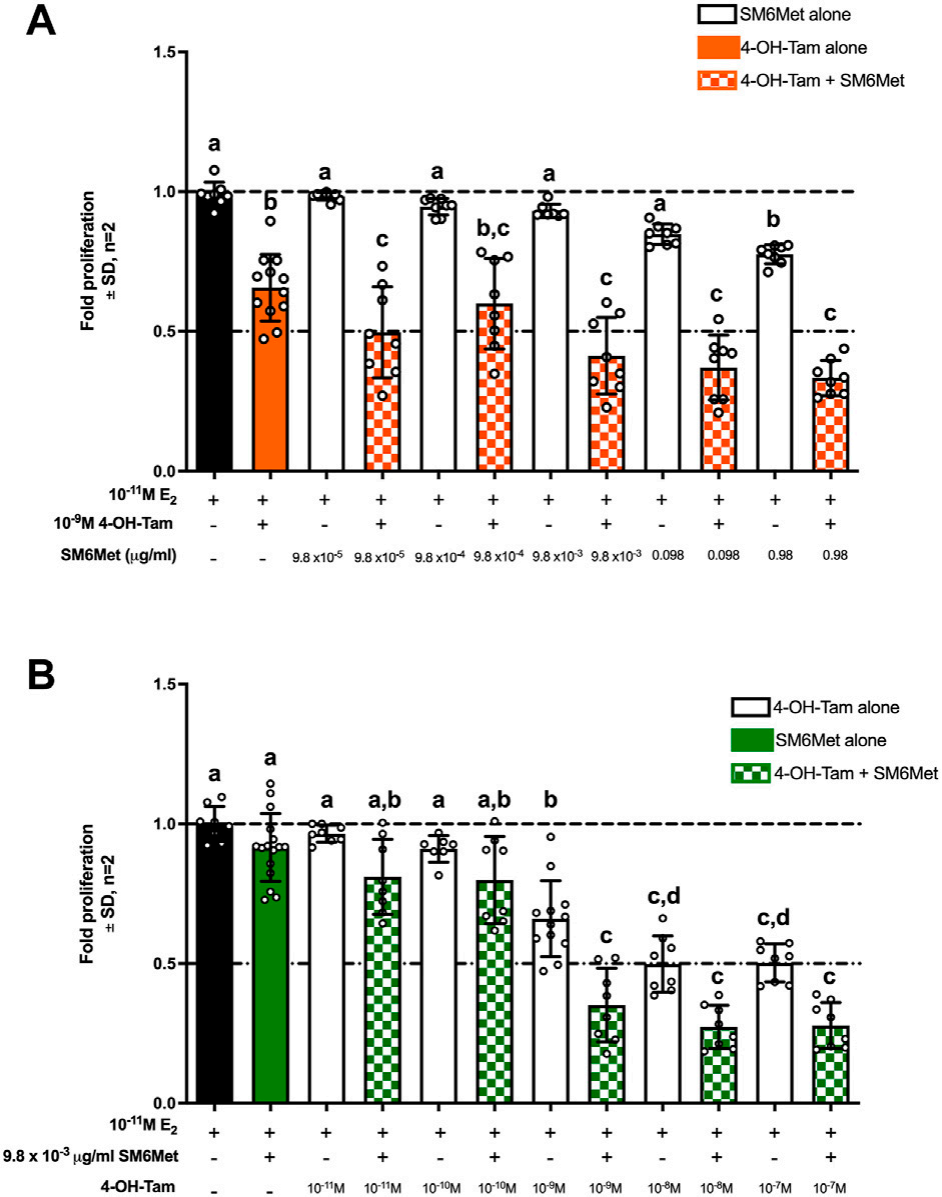
Combinatorial MTT assay for the effect of 4-OH-Tam combined with SM6Met on breast cancer cell proliferation. **(A)** MCF7BUS cells were induced with increasing concentrations of SM6Met combined with a constant concentration of 4-OH-Tam, in the presence of 10^–11^M E_2_, for a period of 7 days, wherein there were two re-treatments. **(B)** MCF7BUS cells were treated with increasing concentrations of 4-OH-Tam combined with a constant concentration of SM6Met, in the presence of 10^–11^M E_2_, for a period of 7 days, wherein there were two re-treatments. Thereafter, viable cells were determined using the MTT assay. Average ± SD is of two independent biological experiments done in quadruplicate. The results were expressed as fold proliferation relative to the positive control, 10^−11^M E_2_, which is set at 1. See Supplementary Figure S2 for results from solvents and E_2_ and Supplementary Figure S3 for a progress curve with E_2_. Statistical analysis was performed using one-way ANOVA analysis of variance with Tukey’s multiple comparisons test as post-test, where different letters indicate statistically significant differences at *p* < 0.05. Bars with common letters are not significantly different.

In Figure 1A, a constant concentration of 4-OH-Tam (10^−9^M) was combined with increasing concentrations of SM6Met. 4-OH-Tam on its own displayed 0.66-fold proliferation which translates to 34% (*p* < 0.001) inhibition of E_2_-induced cell proliferation, while inhibition by the highest concentration (0.98 μg/ml) of SM6Met was only half as effective (0.78-fold proliferation; 22% inhibition, *p* < 0.05). However, when combined with SM6Met (0.0098 μg/ml, 0.098 μg/ml and 0.98 μg/ml), the fold-inhibition of E_2_-induced cell proliferation was significantly increased compared to 4-OH-Tam alone. The higher the concentration of SM6Met in the combination, the higher the inhibition of E_2_-induced ER^+^ breast cancer cell proliferation. The most effective combination (0.98 μg/ml SM6Met with 10^−9^M 4-OH-Tam) displayed 0.33-fold proliferation which translates to 67% inhibition of E_2_-induced cell proliferation, which is 33% higher than the efficacy of 10^−9^M 4-OH-Tam alone and 45% higher than the efficacy of 0.98 μg/ml SM6Met alone.

In Figure 1B, a constant concentration of SM6Met (0.0098 μg/ml) was combined with increasing concentrations of 4-OH-Tam. SM6Met at 0.0098 μg/ml could not significantly inhibit (0.91-fold proliferation; 9% inhibition) E_2_-induced cell proliferation on its own, while 4-OH-Tam significantly reduced E_2_-induced cell proliferation in a dose dependent manner with significant inhibition at concentrations of 10^−9^M (0.66-fold proliferation; 44% inhibition, *p* < 0.001), 10^−8^M (0.5-fold proliferation; 50% inhibition, *p* < 0.001) and 10^−7^M (0.5-fold proliferation; 50% inhibition, *p* < 0.001). However, when the 0.0098 μg/ml SM6Met was combined with 4-OH-Tam, the fold-inhibition of E_2_-induced cell proliferation increased in relation to the increase in concentration of 4-OH-Tam. The most effective combination (10^−8^M 4-OH-Tam with 0.0098 μg/ml SM6Met) displayed 0.27-fold proliferation which translates to 73% inhibition, which is 23% higher than the efficacy of 10^−8^M 4-OH-Tam alone and 64% higher than the efficacy of 0.0098 μg/ml SM6Met alone.

### 3.2 Synergistic effect of SM6Met and 4-OH-Tam in attenuating E_2_-induced ER^+^ breast cancer cell proliferation

**FIGURE 2:**
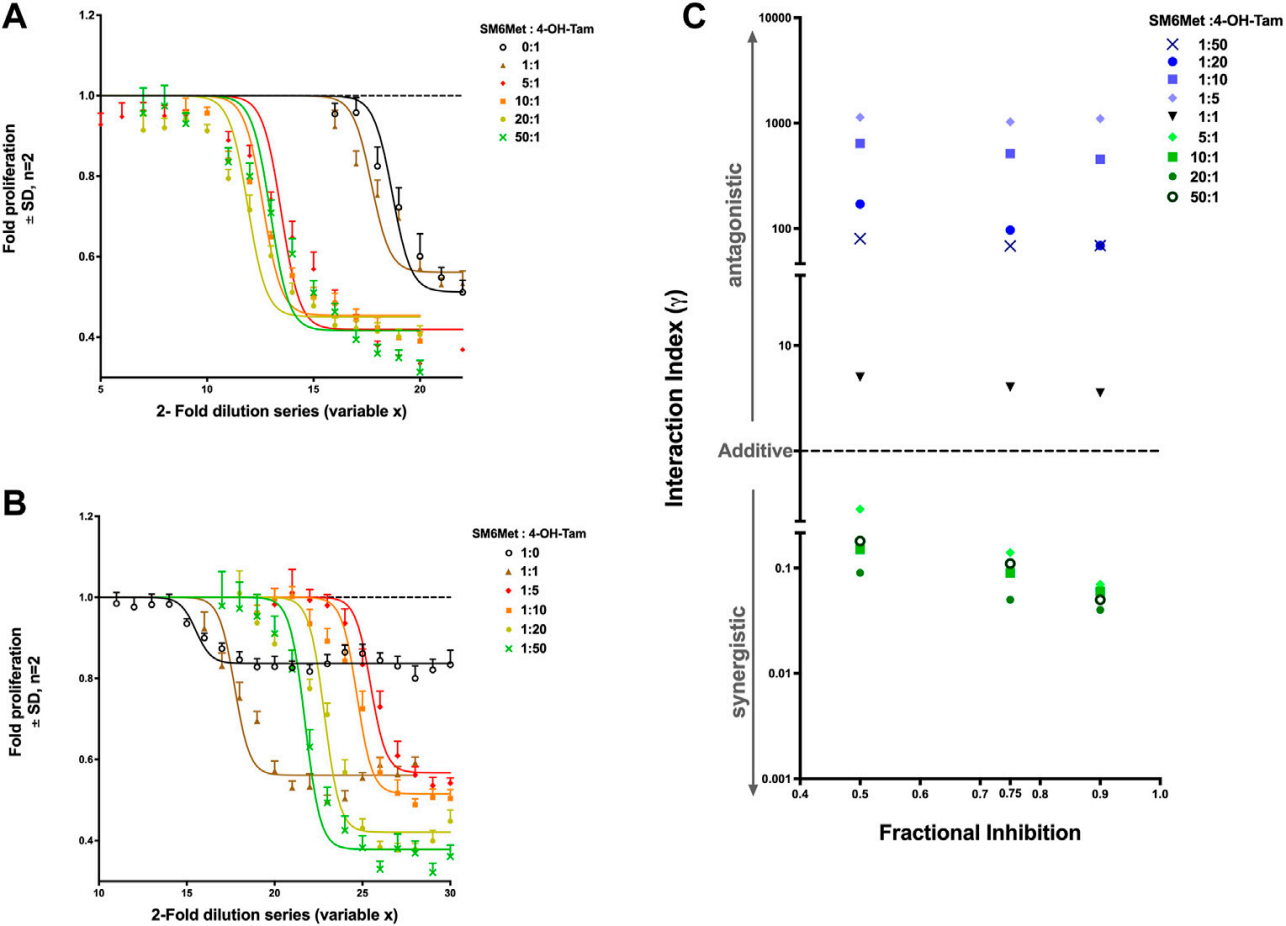
Synergistic effect of SM6Met and 4-OH-Tam in attenuating E_2_-induced breast cancer cell proliferation. MCF-7BUS cells were treated with combinations with a higher ratio towards SM6Met **(A)** and combinations with a higher ratio towards 4-OH-Tam **(B)** using a 2-fold dilution series of each combination ratio in the presence of 10^−11^M E_2_ for a period of 7 days wherein there were two retreatments. Thereafter, the number of viable cells were measured using the MTT assay and dose response curves were generated using non-linear regression curve-fitting, specifically the log(inhibitor) vs. response (three parameters) equation in GraphPad Prism was employed with the top of the curve constrained to 1, to determine the potency and efficacy values of each combination. Average ± SD is of two independent biological experiments done in quadruplicate. The results were expressed as fold proliferation relative to the positive control, 10^−11^M E_2_, which is set at 1. See Supplementary Figure S2 for results from solvents and E_2._ **(C)** Interaction index calculated and plotted for each combination ratio of SM6Met:4-OH-Tam at the 50%, 75% and 90% inhibition level of E_2_-induced breast cancer cell proliferation. If the combination is synergistic, the index will be less than one (γ < 1), if additive it will be equal to one (γ = 1), while if it is antagonistic the index will be greater than one (γ > 1).

Figures 2A,B show the dose-response curves of each combination ratio from which the concentration of each individual drug (summarized in Table 1) can be determined at any effective level to subsequently calculate the interaction index (γ), to determine whether a combination is synergistic, additive or antagonistic. In Figure 2A, the dose-response curve of 4-OH-Tam shifts to the left when combined with increasing SM6Met concentrations, indicating an increase in potency. Interestingly, in Figure 2B the curve of SM6Met shifts to the right (decreasing potency) when combined with 4-OH-Tam at a ratio of 1:1 and 1:5. However, when 4-OH-Tam is increased more than five times relative to SM6Met (1:10, 1:20 and 1:50), the curves shift back in the direction of the monotherapy curve of SM6Met.

There was no statistical difference between the efficacy of 4-OH-Tam alone and the efficacy of 4-OH-Tam in a 1:1 ratio combination with SM6Met (Table 1). However, the combinations with more SM6Met, like the 5:1, 20:1 and 50:1 ratios, displayed significantly (*p* < 0.001) higher efficacies than the 1:1 ratio combination and 4-OH-Tam alone. The addition of 4-OH-Tam to SM6Met in a 1:1 ratio significantly increased the efficacy (*p* < 0.001) in comparison to the efficacy of SM6Met alone (Table 1). The combinations with higher 4-OH-Tam ratios were also significantly more efficacious than the efficacy of SM6Met alone. However, in comparison to the efficacy of the 1:1 combination ratio, there was no significant increase in efficacy for the 1:5 and 1:10 combination ratios, with only the 1:20 and 1:50 combination ratios showing a significant increase in efficacy.

The calculated interaction indices (γ) of all the combination ratios were used to create an interaction index plot, a convenient and simple graphic representation of the interaction indices (Figure 2C). The combinations with a higher ratio towards SM6Met (5:1, 10:1, 20:1 and 50:1) showed an interaction index less than one at all three (50%, 75% and 90% inhibition) selected effect levels, whereas the 1:1 combination of SM6Met and 4-OH-Tam and all the combinations with higher ratios towards 4-OH-Tam (1:5, 1:10, 1:20 and 1:50) displayed an interaction index greater than one at all three selected effect levels. This indicates that increasing the concentration of SM6Met in the combination with 4-OH-Tam results in synergy, while increasing the concentration of 4-OH-Tam in the combination results in antagonism. The combinations may be listed in the order of increasing synergism as follows: 5:1 < 50:1 < 10:1 < 20:1 at 50% and 75% inhibition and 5:1 < 10:1 < 50:1 < 20:1 at 90% inhibition. The combinations may be listed in the order of increasing antagonism as follows: 1:1 < 1:50 < 1:20 < 1:10 < 1:5 at 50% and 75% inhibition; and 1:1 < 1:50 = 1:20 < 1:10 < 1:5 at 90% inhibition (Figure 2C). In summary, the combination ratio of SM6Met:4-OH-Tam of 20:1 displayed the lowest interaction index at all the effect levels, thereby making it the combination ratio with the highest degree of synergism.

### 3.3 MCF-7BUS cells accumulate both in the S- and apoptotic-phase in response to co-treatment with SM6Met and 4-OH-Tam

**FIGURE 3:**
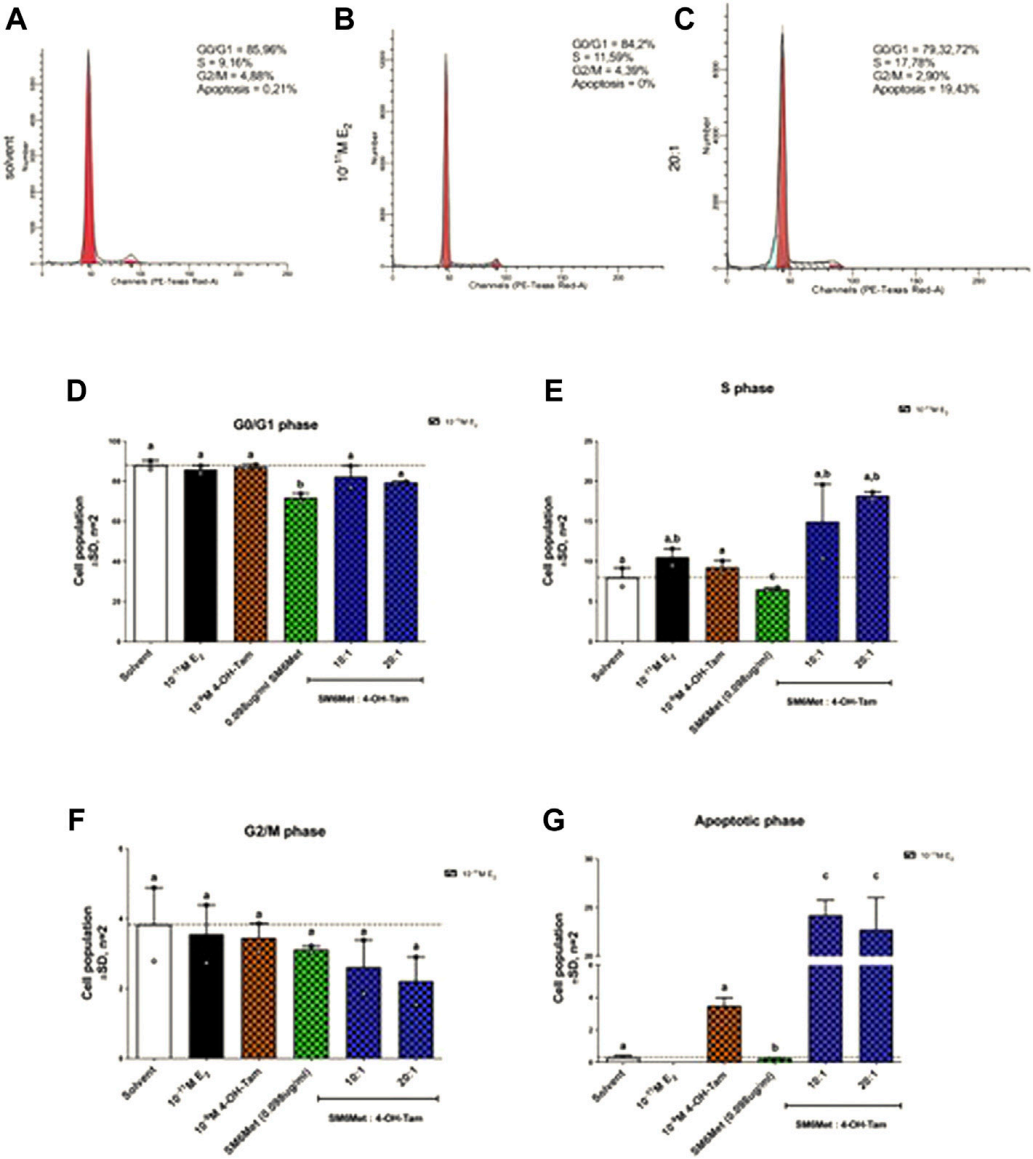
Cell cycle analysis of MCF-7BUS cells shows increased accumulation of cells in the S phase and apoptotic phase in response to increasing amounts of SM6Met in the presence of 4-OH-Tam. Representative histograms of **(A)** solvent, **(B)** 10^−11^M E_2_ and **(C)** the 20:1 combination is shown. The average effect of 4-OH-Tam (10^−9^M), SM6Met (0.098 μg/ml), and combinations of SM6Met with 4-OH-Tam in ratios of 10:1 and 20:1, in the presence of 10^−11^M E_2_ after a 48 h treatment on cells in the **(D)** G_0_/G_1_ phase, **(E)** S phase, **(F)** G_2_/M phase, and **(G)** apoptotic phase is presented as fold change relative to solvent. The dotted line through the bars represents the solvent control, which was set to one. Average ± SD is of two independent biological experiments. Statistical analysis was performed using one-way ANOVA with Tukey’s multiple comparisons test as post-test, where different letters indicate statistically significant differences at *p* < 0.05. Bars with common letters are not significantly different.

Having shown that combining SM6Met with 4-OH-Tam not only resulted in a greater reduction of E_2_-induced ER^+^ breast cancer cell proliferation (Figure 1), but also that SM6Met synergistically enhanced the potency of 4-OH-Tam to reduce ER^+^ breast cancer cell proliferation (Figure 2), the mechanism whereby this occurred was of interest. As proliferation is dependent on the controlled progression of cells through the cell cycle, a process dysregulated in cancer cells to gain infinite replicative potential, we evaluated the effect of the 20:1 and 10:1 combinations of SM6Met with 4-OH-Tam on cell cycle distribution, in the presence of E_2_ (Figure 3). Addition of SM6Met to 4-OH-Tam, in the presence of 10^−11^M E_2_, resulted in a dose dependant increase (1.6-fold increase for the 10:1 ratio and a significant 2.0-fold increase for the 20:1 ratio) in the number of cells in the S phase (Figure 3E) and a dose dependant, although not significant, decrease in the number of cells in the G2/M phase (Figure 3F). Furthermore, the addition of SM6Met to 4-OH-Tam showed a significant (*p* < 0.001) increase (6.9-fold for the 10:1 ratio and 6.4-fold for the 20:1 ratio) in the number of cells in the apoptotic phase in relation to cells treated only with 4-OH-Tam (Figure 3G).

### 3.4 Combining SM6Met with tamoxifen in a ratio of 20:1 inhibited all three processes implicated in ER^+^ breast cancer metastasis to a greater extent than that of the standard of care therapy, 4-OH-Tam, alone

Metastasis to distant organs is the leading cause of death amongst breast cancer patients, accounting for about 90% of breast cancer fatalities (Glück, 2007; Rosa Mendoza et al., 2013; Medeiros and Allan, 2019; Piñeiro et al., 2020). The process of tumour metastasis, collectively known as the metastatic cascade, involves a chain of events including detachment of cells from the primary tumour, invasion into local tissue, intravasation (migration into the blood stream), survival in circulation, extravasation (exit of tumour cells from circulation) and colonization of tumour cells that leads to the formation of a tumour at a secondary site (Valastyan and Weinberg, 2011; Fares et al., 2020). Therefore, evaluating the effect of SM6Met in combination with 4-OH-Tam could provide insight into its ability to treat or prevent ER^+^ breast cancer metastasis.

#### 3.4.1 SM6Met with 4-OH-Tam in a ratio of 20:1 reduced E_2_-induced cell migration

**FIGURE 4:**
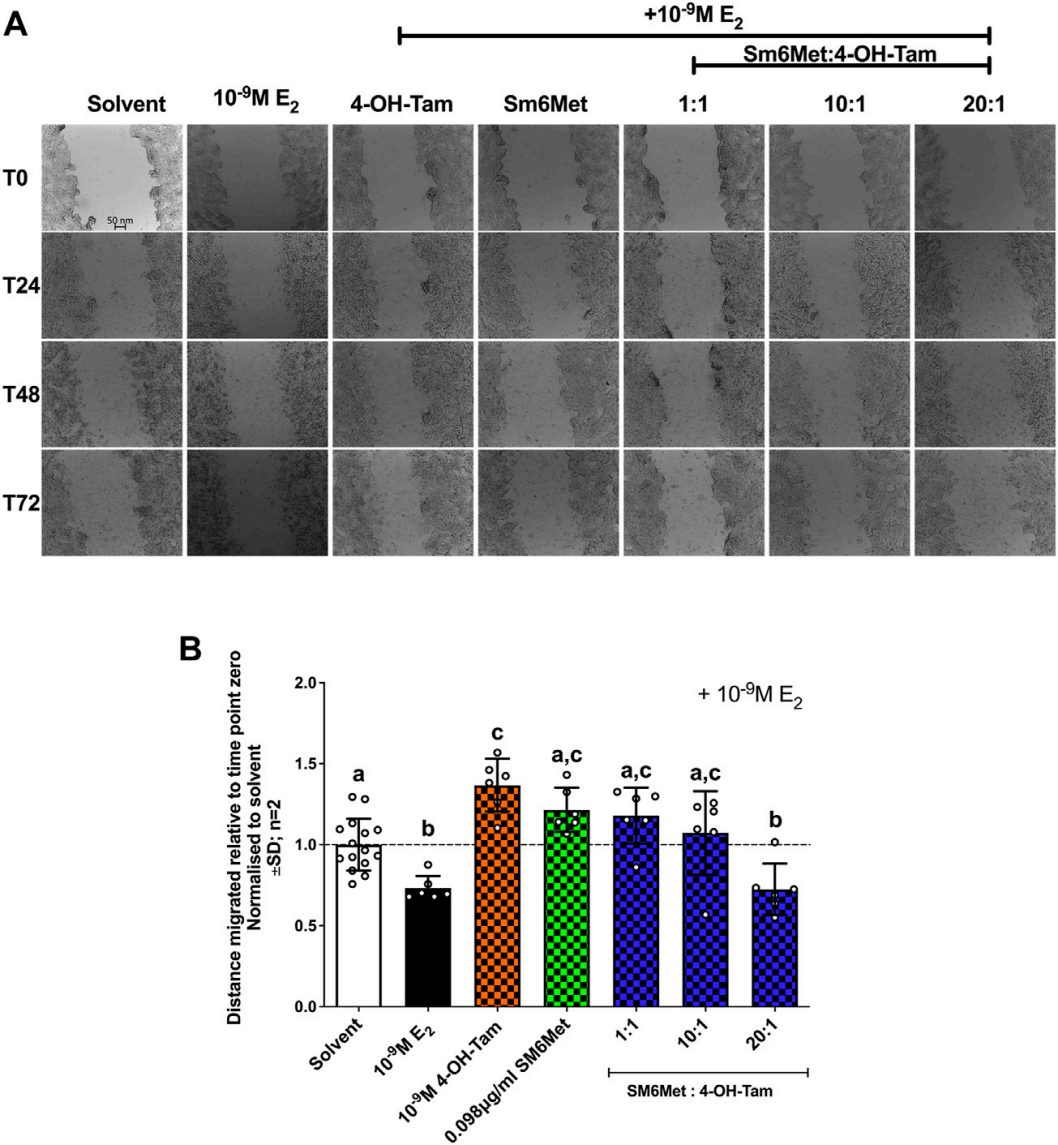
Increasing the concentration of SM6Met in the combinatorial ratio with 4-OH-Tam reduced breast cancer cell migration. **(A)** Directly after treatment, time point zero (T_0_), the scratch wounds were captured using an Olympus IX81 widefield microscope at 10x magnification in 24 h intervals, starting at T_0_ and ending after 72hrs (T_72_). **(B)** The distance migrated was calculated using the formula T_72_-T_0_/T_0_ for each compound or extract and normalised to solvent. The dotted line through the bars represents the solvent, which was set to one. Average ± SD is of two independent biological experiments done in triplicate. Statistical analysis was performed using one-way ANOVA with Tukey’s multiple comparisons test as post-test, where different letters indicate statistically significant differences at *p* < 0.05. Bars with common letters are not significantly different.

Induction with E_2_ (10^−9^M) significantly (*p* < 0.01) decreased ER^+^ breast cancer cell migration (Figures 4A,B), while 4-OH-Tam and SM6Met alone both counteracted the inhibitory effects of E_2_, by significantly increasing cell motility. Interestingly, when 4-OH-Tam was combined with SM6Met it resulted in significant, dose dependant decrease in ER^+^ breast cancer cell migration. At a ratio of 20:1 this reduction was similar to the level of inhibition produced by 10^−9^M E_2_ on its own. Essentially the migratory effects of 4-OH-Tam was reversed when combined with SM6Met, leading to an overall reduction in migration.

#### 3.4.2 SM6Met in combination with 4-OH-Tam is more effective at reducing the number of invasive cells in response to E_2_ than 4-OH-Tam on its own

**FIGURE 5:**
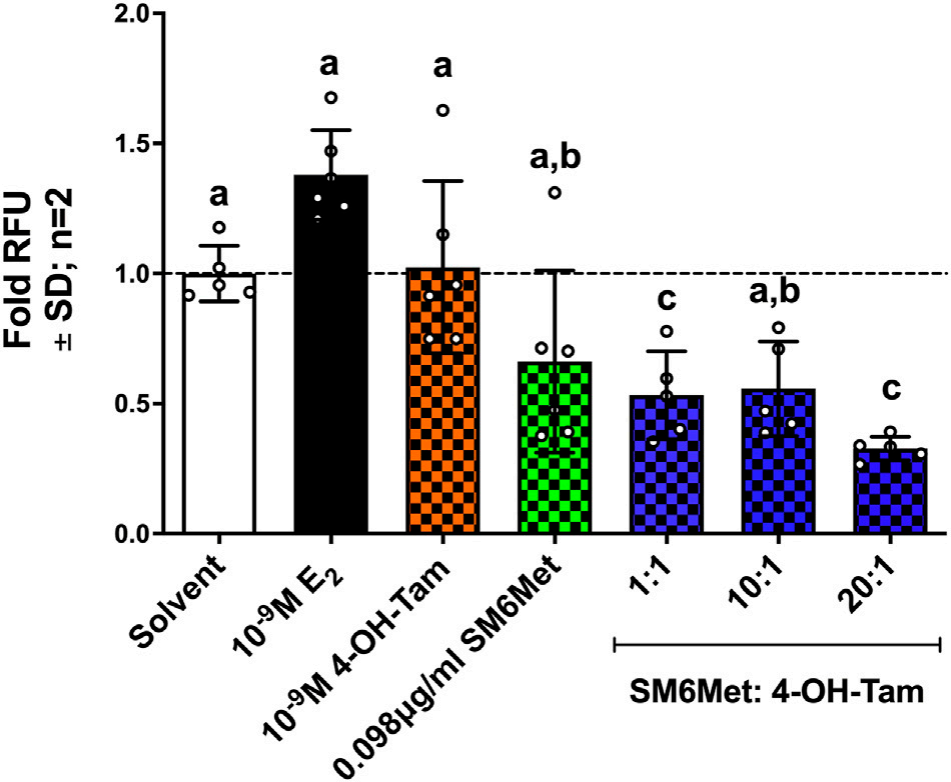
The combination of SM6Met with 4-OH-Tam is more effective than either alone at reducing the number of estrogen-induced invasive breast cancer cells. The number of invasive MCF-7BUS cells was determined using the CytoSelectTM 96-Well cell invasion assay kit. The effects of 4-OH-Tam and SM6Met alone and in combination (1:1, 10:1 and 20:1) on the number of invasive breast cancer cells in the presence of 10^−9^M E_2_ is shown. The dotted line through the bars represents the solvent, which was set to one. Average ± SD is of two independent biological experiments done in triplicate. Statistical analysis was performed using One-way ANOVA with Tukey’s multiple comparisons test as post-test, where different letters indicate statistically significant differences at *p* < 0.05. Bars with common letters are not significantly different.

A significant (*p* < 0.001) increase in the number of invasive ER^+^ breast cancer cells is observed after induction with 10^−9^M E_2_ (Figure 5). 4-OH-Tam shows a non-significant 1.3-fold (25.7%) and SM6Met a significant 2.1-fold (52%) reduction of E_2_-induced ER^+^ breast cancer cell invasion. The addition of SM6Met to 4-OH-Tam at combination ratio’s 1:1 and 1:20, resulted in a further significant (*p* < 0.05) increase in the reduction of ER^+^ breast cancer cell invasion as compared to 4-OH-Tam or SM6Met alone, with the highest inhibition elicited by the 20:1 combination ratio.

#### 3.4.3 SM6Met in combination with 4-OH-Tam is more effective at inhibiting E_2_-induced colony formation than 4-OH-Tam on its own

**FIGURE 6:**
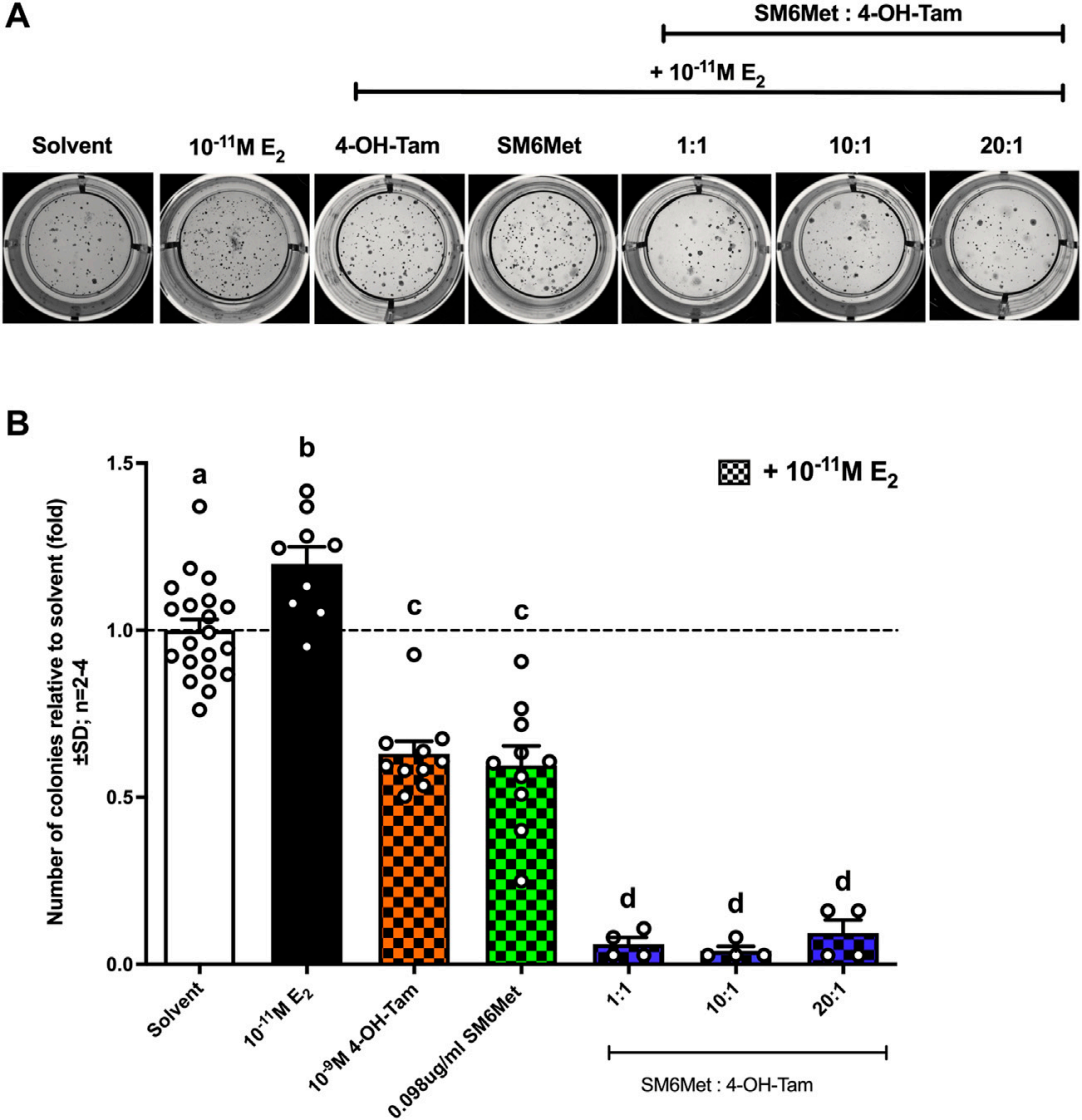
All combinations of SM6Met with 4-OH-Tam showed greater inhibition of colony formation than SM6Met and 4-OH-Tam on their own. MCF7-BUS cells were suspended in 0.6% agarose and added onto the bottom 1% agarose layer. The cells were treated weekly with 4-OH-Tam and SM6Met alone or in combination (1:1, 10:1 and 20:1), all in the presence of 10^−11^M E_2_ for the duration of 21 days. **(A)** Representative images of colonies when induced with compound or extract in the presence of 10^−11^M E_2_ were taken on day 21 after first treatment. **(B)** The number of colonies formed were counted using ImageJ software and data represented as fold relative to the average results from the three negative solvent controls including (1) treatment medium, (2) 0.1% (v/v) EtOH in treatment medium and (3) 0.025% (v/v) DMSO in treatment medium. The dotted line through the bars represents the solvent, which was set to one. Average ± SD is of at least two independent biological experiments done in triplicate. Statistical analysis was performed using One-way ANOVA with Tukey’s multiple comparisons test as post-test, where different letters indicate statistically significant differences at *p* < 0.05. Bars with common letters are not significantly different.

SM6Met (in the presence of 10^−11^M E_2_) displayed a similar level of inhibition of colony formation as 4-OH-Tam (Figures 6A,B), however, when SM6Met was combined with 4-OH-Tam in a 1:1 ratio it resulted in a significant (*p* < 0.001) 10-fold further reduction of colony formation. The level of inhibition, however, did not significantly change when the concentration of SM6Met was increased in the combination ratio with 4-OH-Tam suggesting that the 1:1 combination ratio is sufficiently efficient at reducing colony formation and that higher concentrations of SM6Met are not required.

## 4 Discussion

### 4.1 Combinatorial treatment of SM6Met and 4-OH-Tam is significantly more effective at inhibiting E_2_-induced ER^+^ breast cancer cell proliferation than 4-OH-Tam alone

Combination studies aim to reduce the amount of the drug needed to elicit a desired response, thereby, potentially reducing adverse side-effects, and overcoming resistance (Ziauddin et al., 2014; AlFakeeh and Brezden-Masley, 2018; Luque-Bolivar et al., 2020). Generally, this multi-drug approach is used in cancer therapy to target alternative signalling pathways from those used by current SOC therapies in an attempt to delay resistance to the individual drugs (Banerjee et al., 2008; Gandhi et al., 2015; Samadi et al., 2015). However, despite the fact that current SOC endocrine therapies mainly target the ER, the ER remains a viable target after the onset of resistance to SOC endocrine therapy (Riggins et al., 2007; Rondón-Lagos et al., 2016; Luque-Bolivar et al., 2020; Yao et al., 2020), suggesting that the addition of another ER-targeted anti-cancer agent, such as SM6Met (Visser et al., 2013; Oyenihi et al., 2018), in combination with current SOC endocrine therapies, like tamoxifen, could prove effective for overcoming breast tumour resistance to tamoxifen.

Using the combinatorial MTT assay, which is similar to the checkerboard assay, the most commonly used method in antibiotic, anti-microbial, immune disease and viral infection studies to validate improved effectiveness of a drug combination (veldstra, 1956; White et al., 1996; Doern, 2014), the current study showed that the degree of inhibiting E_2_-induced ER^+^ breast cancer cell proliferation was increased by combining 4-OH-Tam with SM6Met. However enhanced effectiveness does not necessarily mean that the drug combination is synergistic. In contrast to the combinatorial MTT assay that only measures enhanced or reduced efficacy, methods to determine synergism measure the degree of enhancement or reduction by the change in potency.

Using the fixed ratio isobolagram method to determine the interaction index (γ) as described by Tallarida *et al.* (Tallarida, 1992; Tallarida et al., 1997) the current study demonstrates for the first time that the *C. subternata* extract, SM6Met, synergistically promotes tamoxifen-induced antagonism of E_2_-induced ER^+^ breast cancer proliferation. Specifically, SM6Met in a ratio combination of 20:1 with 4-OH-Tam produced the lowest interaction index (γ50 of 0.09, γ75 of 0.05 and γ90 of 0.04) and therefore, the highest degree of synergism. We used the interaction index plot (Figure 2C) instead of the conventional isobolagram to simplify the graphical representation of the data in addition to showing the relationship between the different effect levels. In regard to anti-cancer therapies, synergism (γ < 1) at high effect levels is more advantageous than synergism at low effect levels. For example, a combination therapy that is synergistic at the low effect level of 50% means that only 50% of the concentration range used in the dose response curve will be synergistic, whereas a combination therapy that is synergistic at a high effect level of 90% will be synergistic at 90% of the concentration range of the dose response curve (Chou and Talalay, 1984; Chou, 2006). Therefore, the fact that the combination ratio of 20:1 SM6Met:4-OH-Tam not only has the lowest interaction index, but that it is synergistic at all the effect levels (50%, 75% and 90%) tested is propitious.

Other studies have also reported synergistic effects of plant-derived products in combination with 4-OH-Tam. Examples include studies by Yaacob et al. (2014), Khamis et al.(2018), and Kim et al. (2020) which used the Chou-Talalay non-constant ratio drug combination method to determine synergism, and others such as by Chisholm et al. (2004), Samadi et al. (2014) and Blasco-Benito et al. (2018) that did not. Yaacob et al. (2014), showed synergistic inhibition of MCF-7 and MDA-MB-231 breast cancer cell growth by the combination of a bioactive subfraction of *Strobilanthes crispus* leaves (SCS—a shrub originally from Madagascar) and tamoxifen with combination index (CI) values of 0.32–0.40 for MCF-7 cells and 0.29–0.52 for MDA-MB-231 cells at 84–97% effect levels. Khamis et al.(2018), showed synergistic inhibition of MCF7 and T47D ER^+^ breast cancer cell proliferation for all combinations of 4-OH-Tam with Hesperidin (Hes), piperine (Pip) and bee venom (BV). For the MCF7 cell line, the combinations of Tam + Pip, Tam + Pip + BV, and Tam + Hes + Pip + BV had CI values of 0.279, 0.281 and 0.279, respectively and were among the five lowest combination index values. Similarly, for the T47D cell line, the five lowest combination index values were 0.263, 0.315, 0.249, 0.282, and 0.222 for the combinations Tam + Pip, Tam + Hes, Tam + Hes + BV, Tam + Pip + BV and Tam + Hes + Pip + BV, respectively. Kim et al. (2020), showed that ginseng seed oil (GSO) in combination with 4-OH-Tam synergistically inhibits tamoxifen-resistant MCF-7 (MCF-7^TAMR^) ER^+^ breast cancer cell growth with CI values ranging from 0.07 to 0.90. Furthermore, Blasco-Benito et al. (2018), combined Δ^9^-tetrahydrocannabinol (THC) or a cannabis drug preparation (CDP) from Cannabis sativa with tamoxifen and suggest synergistic inhibition of T47D and MCF7 ER^+^ breast cancer cells by the CDP that they attributed to the “entourage effect”. While a study by Chisholm et al. (2004) showed synergistic cytotoxic effects of epigallocatechin gallate (the most common catechin found in green tea) in combination with tamoxifen on MDA-MB-231 breast cancer cells and a study by Samadi et al. (2014) showed synergistic inhibition of proliferation and induction of apoptosis in MDA-MB-231 and H1299 cells by the combination of vinblastine (isolated from the flowering Madagascan plant, Catharanthus roseus) with tamoxifen.

Due to the various theories, hypotheses, approaches, and models used, it is hard to compare the claimed synergistic results of the previously mentioned studies with the results obtained in the current study. Although all three studies claim synergism, the studies by Chisholm et al. (2004), Samadi et al. (2014) and Blasco-Benito et al. (2018) merely indicate enhanced effectiveness for the various combinations and as discussed by Chou and Talalay (1984) and Chou (2006), enhanced effectiveness does not necessarily mean that the drug combination is synergistic. The most recent methods to determine synergy describe synergism as a measure of the degree of enhancement or reduction in potency and not effectiveness (Tallarida, 2002; Chou, 2006). Without a standardized method of analysis, unsubstantiated or faulty claims of synergism are inevitable. Although, the studies by Yaacob et al. (2014), Khamis et al. (2018), and Kim et al. (2020) did establish combination index (CI) values, which is comparable to the interaction index (γ) calculated in the current study, in contrast to the current study, Yaacob et al. (2014) determined it for effect levels 84%–97%, Khamis et al. (2018) only determined it for the 50% effect level, while the effect level in Kim et al. (2020) is not indicated. Nonetheless, the best interaction index value (0.04) achieved in the current study by the 20:1 ratio of SM6Met:4-OH-Tam at the 90% effect level is substantially greater than the best CI values (0.32 and 0.279, respectively) achieved by Yaacob et al. (2014) and Khamis et al. (2018) in the same MCF-7 cell line, while Kim et al. (2020) achieved a best CI value of 0.07 in a tamoxifen-resistant MCF-7 cell line.

Cell cycle analysis sheds some light on the mechanism whereby the combinatorial treatment of SM6Met and 4-OH-TAM could affect MCF-7BUS cell proliferation. Specifically, the addition of SM6Met to 4-OH-Tam in the combination ratio of 20:1 significantly increased the number of cells in the S phase and the number of apoptotic cells, while decreasing the number of cells in the G2/M phase. In contrast the study by Khamis et al. (2018), show for all combinations with tamoxifen (except Tam + Hes) a significant increase in the number of MCF7 and T47D cells in G2/M phase.

Although previous studies have shown that treatment with 4-OH-Tam induced a significant G0/G1 phase arrest (Osborne et al., 1983, 1984; Lykkesfeldt et al., 1984; Yeh et al., 2014; Khamis et al., 2018), the current study does not reflect this, which may be ascribed to the fact that the concentration of 4-OH-Tam used in the current study (10^- 9^M) was much lower than that used in previous studies (10^−3^M–10^−6^M). SM6Met, in the presence of E_2_, however, displayed similar cell cycle distribution patterns as a previous study also using MCF-7BUS ER^+^ breast cancer cells (Visser, 2013) by demonstrating arrest in the S phase. Thus, as 4-OH-Tam has been shown by others (Osborne et al., 1983, 1984; Lykkesfeldt et al., 1984; Yeh et al., 2014; Khamis et al., 2018) to arrest cells in the G0/G1 phase, while SM6Met arrests in the S phase, it implies that SM6Met may elicit its effects on the regulation of cell cycle machinery via a different mechanism to that of the SOC therapy, which is preferred for combination therapies as studies suggest breast cancer is more responsive to combinations that inhibit multiple molecular targets associated with the development and progression of breast cancer (Gandhi et al., 2015; Samadi et al., 2015). Together this suggests that adding SM6Met to 4-OH-Tam mechanistically enhanced S phase arrest, which conceivably lead to morphological changes and a subsequent increase in apoptosis, thereby significantly enhancing the pro-apoptotic effects of 4-OH-Tam.

Like our study, a previous study by Charalambus et al. (2013), showed that the combination of equol, the metabolite of the soy phytoestrogen, diazen, with 4-OH-Tam also significantly enhanced the number of apoptotic cells in comparison to equol and 4-OH-Tam alone through activation of caspase-mediated apoptotic pathways. However, data regarding the molecular mechanism by which SM6Met or other compounds enhance the anti-ER^+^ breast cancer activity of tamoxifen is limited and still largely unknown.

### 4.2 Combining SM6Met with tamoxifen in a ratio of 20:1 inhibited all three processes implicated in ER^+^ breast cancer progression and metastasis to a significantly greater degree than 4-OH-Tam alone

Sustained proliferation is one of the six hallmarks of cancer (Hanahan and Weinberg, 2000, 2011; Dai et al., 2016). Having shown that addition of SM6Met to 4-OH-Tam resulted in synergy of anti-proliferative effects on E_2_-induced ER^+^ breast cancer cell proliferation, we shifted our investigation to evaluate the effects of the combinatorial treatment of SM6Met and 4-OH-Tam on migration, invasion, and colony formation, three processes not only involved in cancer metastasis, but which are also characteristic hallmarks of cancer.

Previous studies have shown that in ER^+^ breast cancer cells E_2_ stimulates migration through activation of mitogen-activated protein kinase (MAPK) phosphorylation of cSRC, which in turn interacts with focal adhesion kinases and the delta 5 truncated form of SRC3. This process stimulates the development of filopodia and pseudopodia at the leading edges of the breast cancer cells (Li et al., 2010; Flamini et al., 2011; Sanchez et al., 2011). However, in contrast to most (Lymperatou et al., 2013; Park et al., 2016), but not all studies (Sisci et al., 2010; Gao et al., 2017; Padilla-Rodriguez et al., 2018), we show that 10^−9^M E_2_ inhibits ER^+^ breast cancer cell migration, which may be attributed to differences in methodology like different cell lines, induction periods, culture conditions and the use of mytomycin C, an inhibitor of cell proliferation used to accurately identify the migratory potential. The use of mitomycin C has only recently been introduced to migratory studies (wound healing assays) to distinguish between actual migration and proliferation. Moreover, no literature was found to support the effects demonstrated by 4-OH-Tam or SM6Met alone, nonetheless, 4-OH-Tam and SM6Met completely reversed the protective effects of E_2_ on ER^+^ breast cancer cell migration. We also show for the first time, that SM6Met in combination with 4-OH-Tam in a ratio of 20:1 inhibits ER^+^ breast cancer cell migration to a level greater than that of either SM6Met or 4-OH-Tam alone.

There is contradicting evidence as to the effects of E2 on ER^+^ breast cancer cell invasion as some studies have shown that E_2_ reduces ER^+^ breast cancer cell invasion (Lymperatou et al., 2013), some show no effect (Thompson et al., 1988; Visser, 2013) and others, like our study, indicate an increase in ER^+^ breast cancer cell invasion (Thompson et al., 1988; Di et al., 2015). Although 4-OH-Tam, in the presence of E_2_, had no significant effect on ER^+^ breast cancer cell invasion in the current study, it has previously been shown that 4-OH-Tam increases ER^+^ breast cancer cell invasion in relation to E_2_ (Thompson et al., 1988; Lymperatou et al., 2013). Furthermore, we also show for the first time that SM6Met, in the presence of E_2_, was able to inhibit ER^+^ breast cancer cell invasion to a greater extent than the SOC therapy, 4-OH-Tam alone and that the addition of SM6Met to 4-OH-Tam, further increased the inhibition of ER^+^ breast cancer cell invasion, to a level beyond that of SM6Met or 4-OH-Tam alone.

In line with previous studies E_2_ displayed an increase in colony formation (Xie et al., 2001; Cui et al., 2006), while 4-OH-Tam inhibited E_2_-induced colony formation (Xie et al., 2001). We show for the first time that SM6Met can inhibit colony formation to the same extent as the SOC therapy, 4-OH-Tam, and that when adding SM6Met to 4-OH-Tam, the inhibitory effects are enhanced. Hence, the combination of SM6Met with 4-OH-Tam was more effective at targeting ER^+^ breast cancer cell migration, invasion, and colony formation than the SOC therapy, 4-OH-Tam, alone. As the combination of SM6Met and 4-OH-Tam was the only treatment to substantially inhibit all three processes implicated in ER^+^ breast cancer metastasis, it shows great potential to not only be developed as treatment for primary or early-stage ER^+^ breast cancer, but also metastatic ER^+^ breast cancer.

## 5 Conclusion

In summary, the results of the current study present insights into the potential of SM6Met as a compliment to current SOC treatment for ER^+^ breast cancer, either as monotherapy or in combination with the current SOC therapy, 4-OH-Tam.

**FIGURE 7:**
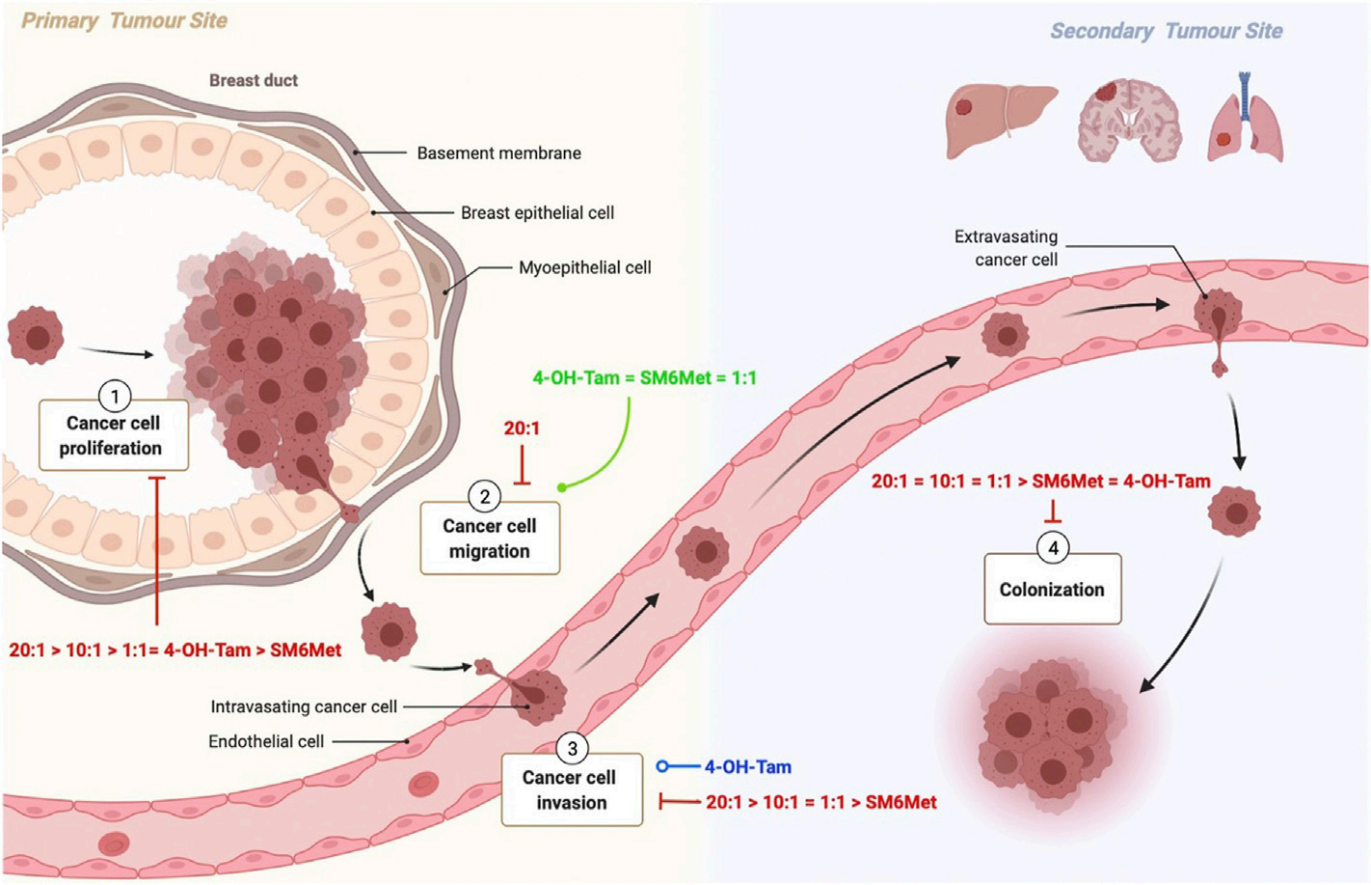
Schematic summary of the effects of the combinations of SM6Met with 4-OH-Tam compared to SM6Met alone and 4-OH-Tam alone on the processes involved in breast cancer proliferation and metastasis. Breast cancer carcinogenesis encompasses various steps, each of which present an opportunity for new therapies. Carcinogenesis is characterized by uncontrolled cell proliferation 1), which leads to the acquisition of specific properties which allow the tumour cell to detach, migrate 2) and invade 3) local tissue to ultimately enter into the circulation, travel to distant organs and form colonies 4) at the secondary tumour site. Although most tumour cells circulate as single cells, others travel as clusters that include stromal cells, neutrophils, and platelets, which is more likely to form metastasis. Here the red solid line indicates the inhibiting effects of the combinations of SM6Met with 4-OH-Tam in comparison to SM6Met alone and 4-OH-Tam alone, all in the presence of E_2_, listed in order of efficacy, while the green solid line represents induction, and the blue line represents no effect.

As monotherapy, SM6Met was able to inhibit three out of the four processes evaluated in this study namely, proliferation, invasion and colony formation (Figure 7). SM6Met, like 4-OH-Tam was able to significantly inhibit E_2_-induced ER^+^ breast cancer cell proliferation, however it could not attain the same potency nor efficacy as the SOC therapy, tamoxifen. SM6Met was more effective or just as effective at inhibiting E_2_-induced ER^+^ breast cancer cell invasion and colony formation, respectively, as the SOC therapy, 4-OH-Tam. Together these results suggest that SM6Met as monotherapy cannot compete with current SOC therapy at targeting ER^+^ breast cancer cell proliferation, however, SM6Met may prove just as effective as the SOC therapy at targeting pro-metastatic processes such as ER^+^ breast cancer cell invasion and colony formation, but not cancer cell migration.

The combination of SM6Met with 4-OH-Tam at a ratio of 20:1 was the only treatment able to significantly inhibit all processes evaluated in this study i.e., proliferation, migration, invasion and colony formation in the presence of E_2_. For the first time we demonstrate that the combination of 4-OH-Tam and SM6Met produces a strong synergistic effect in terms of antagonising E_2_-induced ER^+^ breast cancer cell proliferation. In combination with SM6Met, 20-times lower concentrations of 4-OH-Tam are required to produce the same inhibitory effect on cell proliferation as with 4-OH-Tam alone. Furthermore, increasing the concentration of SM6Met in combination with 4-OH-Tam to a ratio of 20:1 resulted in an overall inhibition of ER^+^ breast cancer cell migration not seen with either 4-OH-Tam or SM6Met alone. This combination of SM6Met with 4-OH-Tam was the only treatment strategy, apart from E_2_, to inhibit ER^+^ breast cancer cell migration. Although 4-OH-Tam, in the presence of E_2_, had no significant effect on ER^+^ breast cancer cell invasion, when added in combination with SM6Met it displayed significant inhibition to a level greater than that of SM6Met alone. Specifically, the 20:1 combination ratio of SM6Met with 4-OH-Tam displayed the highest inhibition of ER^+^ breast cancer cell invasion. Moreover, this combination of SM6Met with 4-OH-Tam also displayed the highest level of inhibition of colony formation, which is significantly greater than observed with either 4-OH-Tam or SM6Met alone. Despite the promising results, to strengthen the conclusions of the current study, future work is needed to validate the findings in additional cell lines and to establish the optimal concentrations of the test compounds and combination ratios of the mixtures evaluated in this study for each process involved in metastasis as the sensitivity of the various assays differ. Furthermore, studies to evaluate the mechanism of action whereby the combinatorial treatment of SM6Met and 4-OH-Tam is superior to that of either agent alone should be considered, as should orthogonal methods for mechanisms, such as apoptosis, explored in the current study.

The results from the current study suggest that the combination of SM6Met with 4-OH-Tam could be a viable drug combination, which may potentially delay resistance and ameliorate the negative side effects associated with tamoxifen monotherapy while, in addition, ultimately inhibiting or preventing metastatic progression of ER^+^ breast cancer. In addition, the lower dose of tamoxifen and the incorporation of a honeybush extract do suggest that this would also be a more affordable alternative to conventional chemotherapy and highlights the potential of honeybush tea to be used as a dietary intervention for the prevention of ER^+^ breast cancer. Thus, combined therapies with a compound or extract with ERβ agonist and ERα antagonist properties such as SM6Met may provide a novel approach for the treatment and or prevention of metastatic ER^+^ breast cancer. These promising effects warrant further investigation into the molecular mechanisms through which SM6Met enhances the effects of 4-OH-Tam and whether SM6Met would have the ability to reverse tamoxifen resistance in MCF7 cells. Despite the novelty of the current findings caution should be exercised in assuming that the promising results of a combination therapy of SM6Met and tamoxifen would necessarily extrapolate to humans (Hartung, 2018; Mattes, 2020).

## Data Availability

The original contributions presented in the study are included in the article/Supplementary Material, further inquiries can be directed to the corresponding author.
