# Supplementary material for: Combinatorial treatments of tamoxifen and SM6Met, an extract from Cyclopia subternata Vogel, are superior to either treatment alone in MCF-7 cells
Source: Front Pharmacol. 2022 Sep 22;13:1017690. doi: 10.3389/fphar.2022.1017690 (PMC9535530; doi:10.3389/fphar.2022.1017690)
Supplement: Supplementary file 1 [file DataSheet1.docx]

Supplementary Material

# Supplementary Data

# Supplementary Figures and Tables

## Supplementary Figures


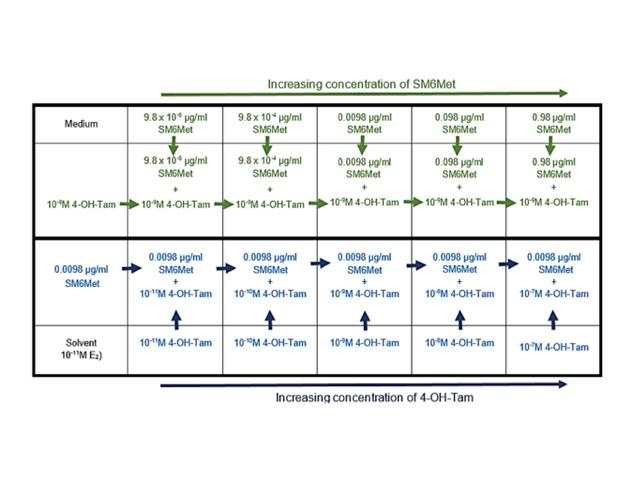


**Supplementary Figure 1:** Diagram depicting the experimental layout for the treatment step of the combinatorial MTT assay, thereby showing how 4-OH-Tam and SM6Met were combined. Increasing concentrations of SM6Met along the horizontal in row one combined with the constant concentration of 4-OH-Tam in row two and increasing concentrations of 4-OH-Tam along the horizontal in row four combined with the constant concentration of SM6Met in row three. Each block represents 4 wells of a 96 well tissue culture plate.

Supplementary Figure 2: Evaluation of the proliferative potential of the controls used in the combinatorial MTT assay. The MCF-7BUS cells were withdrawn from steroids for a week before plating, by changing the growth medium to treatment medium. Thereafter, MCF-7BUS cells were induced with the three negative solvent controls including (1) treatment medium, (2) 0.1% (v/v) EtOH in treatment medium and (3) 0.025% (v/v) DMSO in treatment medium as well as with the positive control, 10^-11^M E_2_ in 0.1% EtOH, for a period of seven days, wherein there were two re-treatments. In addition, the combination of 0.1% (v/v) EtOH and 0.025% (v/v) DMSO was also evaluated as when the test panel samples were added to cells for treatment the final concentration of EtOH did not exceed 0.1% (v/v) and DMSO did not exceed 0.025% (v/v). Thereafter, MTT solution was added to the cells and after a 4-hour incubation the medium was removed and the formazan crystals that formed through metabolism were dissolved in isopropanol. The results were expressed as fold proliferation relative to the medium, which is set at 1. Average ± SD is of two to three independent biological experiments done in duplicate. Statistical analysis was done using one-way ANOVA with Turkey’s multiple comparisons test as post-test, where different letters indicate statistical significance (P<0.05). Bars with common letters are not significantly different.

**
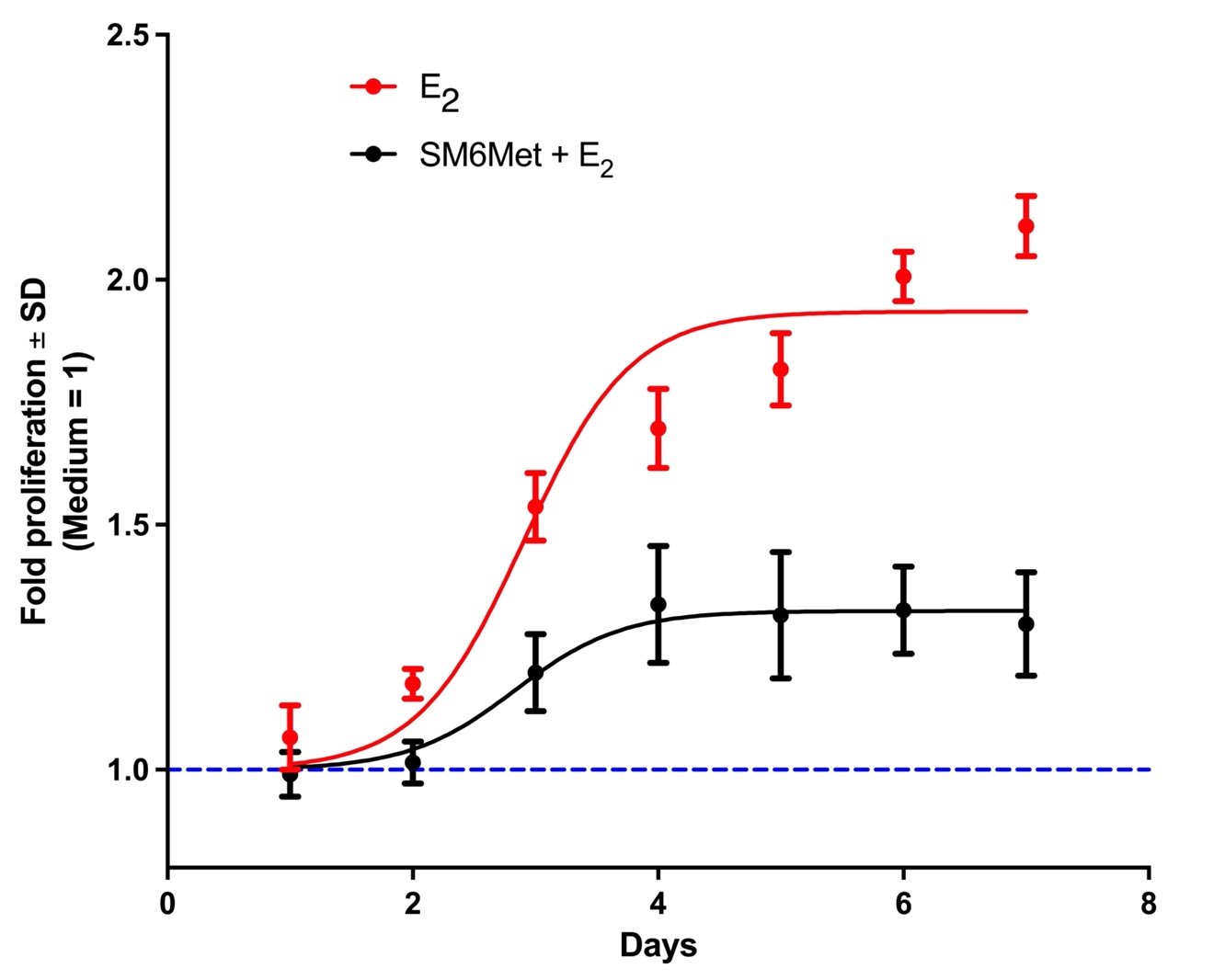
**

**Supplementary Figure 3: Progress curve of the proliferative potential of 10^-11^M E_2_ and 0.098µg/ml SM6Met over a seven-day period**. MCF- 7BUS cells were seeded into 96-well tissue culture plates in treatment medium at a density of 3000 cells/well. The cells were then induced with 10^-11^M E_2_ and 0.098µg/ml SM6Met in the presence of 10^-11^M E_2_ one day after seeding and then two days after first treatment and then two days after second treatment. Proliferation was measured in 24-hour intervals after first treatment for a total of seven days using the MTT assay where MTT solution was added to the cells and after a 4-hour incubation the medium was removed and the formazan crystals that formed through metabolism were dissolved in isopropanol. The results were expressed as fold proliferation relative to the solvent control, which is set at 1 (blue dotted line). Average ± SD is of three independent biological experiments done in duplicate.

## Supplementary Tables

**Supplementary Table 1:** Major polyphenols present in previously prepared *Cyclopia* *subternata* Vogel extract, SM6Met, as determined by HPLC (Visser, 2013).

| Polyphenol | g/100g SM6Met |
| --- | --- |
| Mangiferin | 1.899 |
| Isomangiferin | 0.645 |
| Luteolin | 0.040 |
| Scolymoside (luteolin-7-O- rutinoside) | 1.289 |
| Vicenin-2 (apigenin-6,8-di-C-glucoside) | 0.089 |
| Eriocitrin (eriodictyol-7-O-rutinoside) | 0.846 |
| Hesperidin (hesperitin-7-O-rutinoside) | 2.049 |
| Phloretin-3,5-di-C-glucoside | 1.278 |
| Aspalathin (3-hydroxyphloretin-3', 5'-di-C-hexoside) | 0.700 |
| Iriflophenone-3-C-β-glucoside | 0.669 |
| Iriflophenone-di-O,C-hexoside | 0.958 |
| Protocatechuic acid | 0.113 |
